# Genetic evidence that lower circulating FSH levels lengthen menstrual cycle, increase age at menopause and impact female reproductive health

**DOI:** 10.1093/humrep/dev318

**Published:** 2016-01-04

**Authors:** Katherine S. Ruth, Robin N. Beaumont, Jessica Tyrrell, Samuel E. Jones, Marcus A. Tuke, Hanieh Yaghootkar, Andrew R. Wood, Rachel M. Freathy, Michael N. Weedon, Timothy M. Frayling, Anna Murray

**Affiliations:** Genetics of Complex Traits, University of Exeter Medical School, RILD Level 3, Royal Devon and Exeter Hospital, Barrack Road, ExeterEX2 5DW, UK

**Keywords:** FSH β subunit, menstrual cycle, menopause, endometriosis, fertility

## Abstract

**STUDY QUESTION:**

How does a genetic variant in the *FSHB* promoter, known to alter FSH levels, impact female reproductive health?

**SUMMARY ANSWER:**

The T allele of the *FSHB* promoter polymorphism (rs10835638; c.-211G>T) results in longer menstrual cycles and later menopause and, while having detrimental effects on fertility, is protective against endometriosis.

**WHAT IS KNOWN ALREADY:**

The *FSHB* promoter polymorphism (rs10835638; c.-211G>T) affects levels of *FSHB* transcription and, as a result, circulating levels of FSH. FSH is required for normal fertility and genetic variants at the *FSHB* locus are associated with age at menopause and polycystic ovary syndrome (PCOS).

**STUDY DESIGN, SIZE, DURATION:**

We used cross-sectional data from the UK Biobank to look at associations between the *FSHB* promoter polymorphism and reproductive traits, and performed a genome-wide association study (GWAS) for length of menstrual cycle.

**PARTICIPANTS/MATERIALS, SETTING, METHODS:**

We included white British individuals aged 40–69 years in 2006–2010, in the May 2015 release of genetic data from UK Biobank. We tested the FSH-lowering T allele of the *FSHB* promoter polymorphism (rs10835638; c.-211G>T) for associations with 29, mainly female, reproductive phenotypes in up to 63 350 women and 56 608 men. We conducted a GWAS in 9534 individuals to identify genetic variants associated with length of menstrual cycle.

**MAIN RESULTS AND THE ROLE OF CHANCE:**

The FSH-lowering T allele of the *FSHB* promoter polymorphism (rs10835638; MAF 0.16) was associated with longer menstrual cycles [0.16 SD (*c.* 1 day) per minor allele; 95% confidence interval (CI) 0.12–0.20; *P* = 6 × 10^−16^], later age at menopause (0.13 years per minor allele; 95% CI 0.04–0.22; *P* = 5.7 × 10^−3^), greater female nulliparity [odds ratio (OR) = 1.06; 95% CI 1.02–1.11; *P* = 4.8 × 10^−3^] and lower risk of endometriosis (OR = 0.79; 95% CI 0.69–0.90; *P* = 4.1 × 10^−4^). The FSH-lowering T allele was not associated with other female reproductive illnesses or conditions in our study and we did not replicate associations with male infertility or PCOS. In the GWAS for menstrual cycle length, only variants near the *FSHB* gene reached genome-wide significance (*P* < 5 × 10^−9^).

**LIMITATIONS, REASONS FOR CAUTION:**

The data included might be affected by recall bias. Cycle length was not available for 25% of women still cycling (1% did not answer, 6% did not know and for 18% cycle length was recorded as ‘irregular’). Women with a cycle length recorded were aged over 40 and were approaching menopause; however, we did not find evidence that this affected the results. Many of the groups with illnesses had relatively small sample sizes and so the study may have been under-powered to detect an effect.

**WIDER IMPLICATIONS OF THE FINDINGS:**

We found a strong novel association between a genetic variant that lowers FSH levels and longer menstrual cycles, at a locus previously robustly associated with age at menopause. The variant was also associated with nulliparity and endometriosis risk. These findings should now be verified in a second independent group of patients. We conclude that lifetime differences in circulating levels of FSH between individuals can influence menstrual cycle length and a range of reproductive outcomes, including menopause timing, infertility, endometriosis and PCOS.

**STUDY FUNDING/COMPETING INTEREST(S):**

None.

**TRIAL REGISTRATION NUMBER:**

Not applicable.

## Introduction

FSH is a key pituitary hormone, which stimulates maturation of oocytes and is a biomarker of ovarian reserve. FSH is a heterodimer comprised a hormone-specific β-chain (FSH-β) associated with an α-chain shared by other members of the glycoprotein hormone family (Nagirnaja *et al.*, 2010). The anterior pituitary produces FSH, with transcription of *FSHB* being the rate-limiting step for FSH production. FSH stimulates target cells by binding to the FSH receptor (FSHR), a G-protein-coupled receptor (Fan and Hendrickson, 2005), promoting follicle maturation and estrogen production in women, and Sertoli cell proliferation and spermatogenesis in men (Nagirnaja *et al.*, 2010).

Rare mutations in the *FSHB* gene cause truncation of the FSH-β protein and result in hypogonadism and primary amenorrhoea in females (Layman *et al.*, 1997; Matthews and Chatterjee, 1997; Kottler *et al.*, 2010) and, in a male, delayed puberty with azoospermia (Phillip *et al.*, 1998). Mouse models suggest that FSH is required for normal fertility. Female *Fshb* knockout mice are infertile and fail to complete normal folliculogenesis, while male knockouts remain fertile but have reduced sperm counts, and infertility is observed in both male and female transgenic mice overexpressing human FSH (Kumar *et al.*, 1997, 1999).

A polymorphism in the promoter of *FSHB* (rs10835638; c.-211G>T) −211 bp upstream of the transcription start site is associated with reduced FSH-β production *in vitro* and in human genetic studies. *In vitro*, the T allele of the promoter polymorphism reduces expression of a luciferase reporter gene (Hoogendoorn *et al.*, 2003) and decreases *FSHB* transcription in gonadotroph cells as a result of reduced LHX3 homeodomain transcription factor binding (Benson *et al.*, 2013). The T allele of rs10835638 (c.-211G>T) is associated with lower FSH levels in men and women, and with higher LH and lower testicular volume, sperm count, FSH/LH ratio, inhibin B and testosterone in men, and has been found at a higher prevalence in infertile men (Grigorova *et al.*, 2008, 2010, 2011; Tuttelmann *et al.*, 2012; La Marca *et al.*, 2013; Schuring *et al.*, 2013; Simoni and Casarini, 2014; Ruth *et al.*, 2015). Genetic association studies have identified signals at the *FSHB* locus associated with age at menopause (Stolk *et al.*, 2012; Day *et al.*, 2015), polycystic ovary syndrome (PCOS) (Hayes *et al.*, 2015) and levels of LH (Hayes *et al.*, 2015; Ruth *et al.*, 2015).

Using the unique resource of the UK Biobank (Allen *et al.*, 2014), we show that a common genetic variant known to alter FSH levels impacts a wide range of traits important to female reproductive health, including fertility, endometriosis and menstrual cycle length. In the first genome-wide association study (GWAS) for menstrual cycle length, we identified the *FSHB* locus as the only signal associated with this trait.

## Materials and Methods

### Source of data

The UK Biobank includes data for 503 325 people aged 40–69 years recruited in 2006–2010 from across the UK (Allen *et al.*, 2014). We analysed data from the May 2015 interim release of imputed genetic data from UK Biobank, which contains 73 355 667 single-nucleotide polymorphisms (SNPs), short insertion/deletions and large structural variants in 152 249 individuals [http://www.ukbiobank.ac.uk/wp-content/uploads/2014/04/imputation_documentation_May2015.pdf (17 December 2015, date last accessed)]. UK Biobank invited 9.2 million people to participate, giving a response rate of 5.47% (Allen *et al.*, 2012). Participants were registered with the UK National Health Service and lived within 25 miles of one of the 22 assessment centres. Participants answered detailed questions about themselves, had measurements taken and provided blood, urine and saliva samples. Two arrays with over 95% common marker content were used to genotype the individuals. Approximately 50 000 people were genotyped on the UK BiLEVE array, and the remainder were genotyped on the UK Biobank Axiom array.

### Phenotypes

We derived reproductive phenotypes from the UK Biobank data (Supplementary data). Continuous phenotypes were age at birth of first and last child (females only), age at menarche, age at natural menopause, length of menstrual cycle, number of live births and number of children fathered (included to test the association with male fertility). Menstrual cycle length was only recorded in women who were still cycling and they were asked ‘How many days is your usual menstrual cycle? (The number of days between each menstrual period)’ (excluding those answering <7 or >365; and if the answer was <12 or >60, then the participant was asked to confirm). Cycle length was not available for 25% of women still cycling (1% did not answer, 6% did not know and for 18% cycle length was recorded as ‘irregular’).

To test assumptions of linearity, we analysed the binary outcomes early menarche (lower 5% tail), early menopause (20–44 years), long menstrual cycle (>31 days), short menstrual cycle (≤20 days) and multiple pregnancy loss (>1 case).

We defined two infertility-related binary phenotypes; never pregnant (females) and never fathered a child (males). We analysed female medical conditions as binary outcomes, comparing people reporting a condition (case) with those who did not (control). Medical conditions included dysmenorrhoea, endometriosis, fibroids, irregular menstrual cycles, menopausal symptoms, menorrhagia, ovarian cysts, PCOS, uterine polyps, vaginal/uterine prolapse and breast, endometrial and ovarian cancer. As more general indicators of gynaecological health, we included the medical interventions bilateral oophorectomy or hysterectomy in our analysis.

### Participants

In our analysis, we included individuals who both self-identified as white British and were confirmed as ancestrally Caucasian by UK Biobank from genetic information (*n* = 128 266). We calculated principal components (PCs) for inclusion as covariates in our analyses using FlashPCA (Abraham and Inouye, 2014). PCs were calculated in 120 286 unrelated participants (as identified by UK Biobank) based on 95 535 independent, directly genotyped SNPs (pairwise *r*^2^ < 0.1). These SNPs had a minor allele frequency (MAF) ≥2.5% and missing-ness <1.5% across all participants in the May 2015 interim release of genetic data, and had a Hardy–Weinberg equilibrium (HWE) *P* > 1 × 10^−6^ within the white British participants.

### Testing for associations of the FSHB promoter polymorphism with reproductive phenotypes

We tested the FSH-lowering T allele of the *FSHB* promoter polymorphism (rs10835638; c.-211G>T) for associations with reproductive phenotypes (up to 63 350 women and 56 608 men). SNP rs10835638 was well imputed in the data (imputation quality 0.995; HWE *P* = 0.16; missing rate = 0.3%). All analyses were carried out in males or females as appropriate (based on self-defined sex) using Stata (v13) (StataCorp LP, College Station, TX, USA).

For continuous phenotypes, we transformed the phenotype by adjusting for recruitment centre, age at recruitment and the first five PCs prior to inverse-normalization. We performed linear regression of transformed phenotype on imputed minor-allele dosages at SNP rs10835638 with genotyping chip as a covariate. We carried out a sensitivity analysis of the effect of different transformations, e.g. inverse normalizing the trait prior to calculating the residuals; however, this did not materially affect our results. Since the data on length of menstrual cycle included a wide range of values (Supplementary data, Figs S1 and S2), we carried out analyses on cycles from 21 to 35 days and in women aged <45 and ≥45 years at recruitment. We validated our results for length of menstrual cycle by carrying out analyses in two randomly chosen, equally sized groups. For age at menopause and age at menarche, we also ran analysis using the phenotype definition from the *ReproGen* Consortium GWAS (www.reprogen.org) (untransformed age at menopause between 40 and 60 years not adjusted for age, untransformed age at menarche) to allow comparisons with published data (Stolk *et al.*, 2012; Perry *et al.*, 2014a,b; Day *et al.*, 2015).

For binary outcomes, we performed logistic regression of the phenotype on minor-allele dosages at SNP rs10835638 including the first five PCs, recruitment centre, age at recruitment and genotyping chip as covariates.

### GWAS of length of menstrual cycle

We conducted a GWAS to identify genetic variants associated with length of menstrual cycle (*n* = 9534) using the BOLT-LMM algorithm (described in Loh *et al.*, 2015) from the freely available BOLT-LMM software package [version 2.2, https://data.broadinstitute.org/alkesgroup/BOLT-LMM/ (17 December 2015, date last accessed)] to account for relatedness and population structure. This allowed us to include related individuals who were excluded from the association analysis of the *FSHB* promoter polymorphism (Supplementary data, Table SI)*.* We transformed length of menstrual cycle by adjusting for recruitment centre and age at recruitment prior to inverse-normalization, and performed association testing while adjusting for genotype chip. We filtered results on imputation quality >0.4, HWE *P* > 1 × 10^−5^, and MAF >0.1%, resulting in ∼16.8 million variants that were tested. As the UK Biobank GWAS included more variants than a standard GWAS and we did not have a replication sample available, we chose a threshold of *P* < 5 × 10^−9^, based on a Bonferroni correction for the number of variants tested, rather than the conventional *P* < 5 × 10^−8^.

## Results

### A common allele in the FSHB gene, known to lower FSH levels, is associated with longer length of menstrual cycle

**Figure 1:**
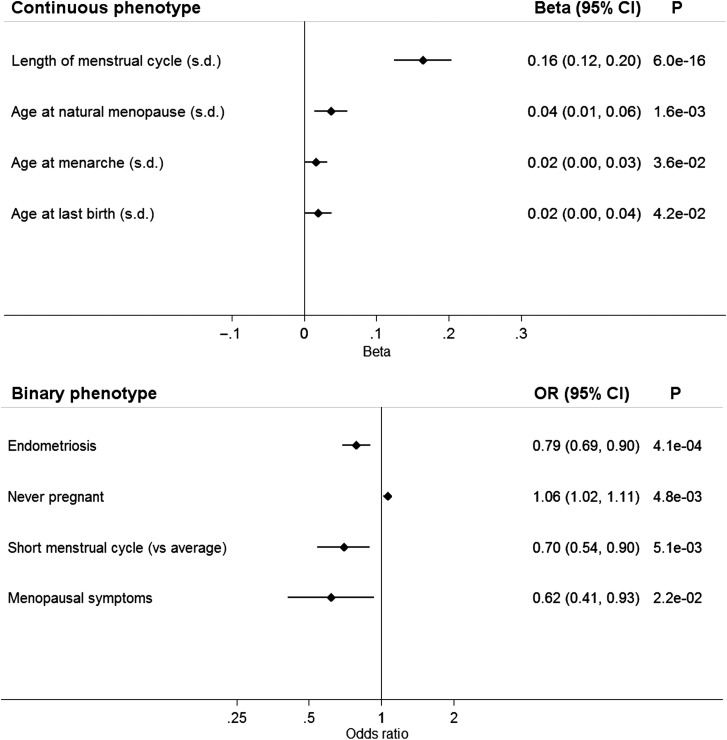
Forest plot of phenotypes associated (*P* < 0.05) with the FSH-lowering T allele of rs10835638 (c.-211G>T). For continuous variables, effects (β) are in standard deviations of the inverse-normally transformed variable to enable effect size comparisons. CI, confidence interval; OR, odds ratio.

The FSH-lowering T allele of the *FSHB* promoter polymorphism (rs10835638; MAF 0.16) was associated with longer menstrual cycles [0.16 SD (∼1 day) per minor allele; 95% confidence interval (CI) 0.12–0.20; *P* = 6 × 10^−16^]. Of the reproductive traits tested (Tables I and II), length of menstrual cycle was the most strongly associated with rs10835638 (Fig. 1 and Table III). The SNP was also associated with cycle length when we dichotomized data into women reporting a cycle length of ≤20 days compared with those reporting an average length of 28 days [odds ratio (OR) = 0.70; 95% CI 0.54–0.90; *P* = 5.1 × 10^−3^] (Fig. 1). There was no evidence for an association with a cycle >31 days compared with the average (OR = 1.16; 95% CI 0.92–1.47; *P* = 0.21). Results remained consistent when we analysed cycle lengths of 21–35 days and when we split our analysis into women aged <45 or ≥45 years (Supplementary data, Fig. S3). Analysis after randomly dividing the sample into two equal parts supported these results (Supplementary data, Fig. S3).

**Table I:** Description of cohort of unrelated individuals for continuous outcome measures.

Phenotype	*n*	Min	Max	Mean	SD	Lower quartile	Median	Upper quartile
Age at first birth (years)^1^	43 066	10	50	25.1	4.6	22	25	28
Age at last birth (years)^1^	43 008	15	50	30.0	4.8	27	30	33
Age at menarche (years)^1^	61 306	9	17	12.9	1.6	12	13	14
Age at natural menopause (years)^1^	27 996	18	65	49.9	4.5	48	50	53
Length of menstrual cycle (days)^1^	8870	7	300	26.8	6.2	25	28	28
Number of children fathered^2^	56 508	0	28	1.8	1.2	1	2	2
Number of live births^1^	63 306	0	22	1.8	1.2	1	2	2

Min, minimum; Max, maximum.

^1^Females only.

^2^Males only.

**Table II:** Number of people included in binary outcome measures.

Phenotype	Description	Cases	Controls	*n*
Bilateral oophorectomy^1^	Yes versus no	5118	57 177	62 295
Dysmenorrhoea^1^	Yes versus none recorded	78	63 272	63 350
Breast cancer^1^	Breast cancer recorded on cancer registry versus none recorded	2810	60 540	63 350
Early menarche^1^	Youngest 5% age at menarche versus oldest 5%	3050	3050	6100
Early menopause^1^	Natural menopause at 20–45 versus 50–60 years	3058	17 805	20 863
Endometrial cancer^1^	Endometrial cancer recorded on cancer registry versus none recorded	342	63 008	63 350
Endometriosis^1^	Yes versus none recorded	993	62 357	63 350
Fibroids^1^	Yes versus none recorded	1819	61 531	63 350
Hysterectomy^1^	Yes versus no	4753	50 932	55 685
Irregular menstrual cycles^1^	Irregular menstrual cycles versus regular cycle	2490	10 316	12 806
Long menstrual cycle (versus average)^1^	Menstrual cycle >31 versus 28 days	237	3889	4126
Menopausal symptoms^1^	Yes versus none recorded	126	63 224	63 350
Menorrhagia^1^	Yes versus none recorded	348	63 002	63 350
Multiple pregnancy loss^1^	More than one pregnancy loss versus none	4047	33 191	37 238
Never fathered child^2^	Never fathered a child versus one or more children fathered	11 729	44 779	56 508
Never pregnant^1^	Never pregnant versus one or more pregnancies	9247	52 966	62 213
Ovarian cancer^1^	Ovarian cancer recorded on cancer registry versus none recorded	247	63 103	63 350
Ovarian cysts^1^	Yes versus none recorded	1 015	62 335	63 350
Polycystic ovary syndrome^1^	Yes versus none recorded	153	63 197	63 350
Short menstrual cycle (versus average)^1^	Menstrual cycle ≤20 versus 28 days	288	3889	4 177
Uterine polyps^1^	Yes versus none recorded	359	62 991	63 350
Vaginal/uterine prolapse^1^	Yes versus none recorded	653	62 697	63 350

^1^Females only.

^2^Males only.

**Table III:** Associations with the FSH-lowering T allele of rs10835638 (c.-211G>T).

Phenotype	Statistic	Effect(95% CI)	SE	*P*-value
Length of menstrual cycle (SD)	β	0.16 (0.12, 0.20)	0.02	**6.0E−16**
Endometriosis	OR	0.79 (0.69, 0.90)	0.05	4.1E−04
Age at natural menopause (SD)	β	0.04 (0.01, 0.06)	0.01	1.6E−03
Never pregnant	OR	1.06 (1.02, 1.11)	0.02	4.8E−03
Short menstrual cycle (versus average)	OR	0.70 (0.54, 0.90)	0.09	5.1E−03
Menopausal symptoms	OR	0.62 (0.41, 0.93)	0.13	2.2E−02
Age at menarche (SD)	β	0.02 (0.00, 0.03)	0.01	3.6E−02
Age at last birth (SD)	β	0.02 (0.00, 0.04)	0.01	4.2E−02
Age at first birth (SD)	β	0.02 (0.00, 0.03)	0.01	7.9E−02
Number of live births (SD)	β	−0.01 (−0.03, 0.00)	0.01	8.1E−02
Never fathered a child	OR	1.03 (0.99, 1.08)	0.02	1.2E−01
Early menopause	OR	0.95 (0.88, 1.02)	0.04	1.6E−01
Early menarche	OR	0.94 (0.85, 1.04)	0.05	2.1E−01
Fibroids	OR	0.94 (0.86, 1.03)	0.04	2.1E−01
Long menstrual cycle (versus average)	OR	1.16 (0.92, 1.47)	0.14	2.1E−01
Polycystic ovary syndrome	OR	1.18 (0.88, 1.59)	0.18	2.7E−01
Ovarian cysts	OR	0.94 (0.83, 1.07)	0.06	3.6E−01
Number of children fathered (SD)	Beta	0.01 (−0.01, 0.02)	0.01	4.1E−01
Menorrhagia	OR	0.92 (0.74, 1.13)	0.10	4.2E−01
Irregular menstrual cycles	OR	0.97 (0.89, 1.06)	0.04	4.6E−01
Multiple pregnancy loss	OR	0.98 (0.91, 1.04)	0.03	4.6E−01
Dysmenorrhoea	OR	0.87 (0.56, 1.38)	0.20	5.6E−01
Breast cancer	OR	1.02 (0.95, 1.10)	0.04	6.4E−01
Ovarian cancer	OR	0.94 (0.74, 1.21)	0.12	6.4E−01
Vaginal/uterine prolapse	OR	0.97 (0.83, 1.13)	0.08	6.7E−01
Uterine polyps	OR	0.98 (0.80, 1.20)	0.10	8.6E−01
Endometrial cancer	OR	1.00 (0.81, 1.23)	0.11	9.7E−01

*Note*: For continuous variables, effects (β) are in standard deviations of the inverse-normally transformed variable to enable effect size comparisons. Results significant at *P* < 5E−08 are in bold; results significant at *P* < 5E−02 are underlined.

CI, confidence interval; OR, odds ratio; SD, standard deviations.

**Figure 2:**
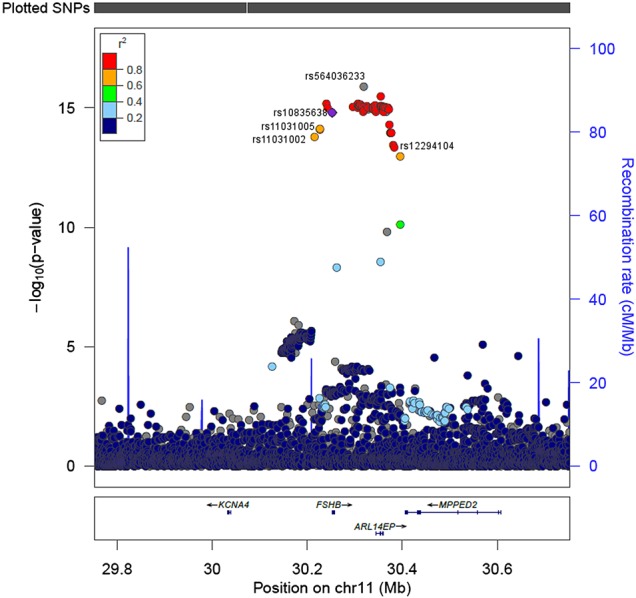
LocusZoom plot showing variants associated with length of menstrual cycle. The most strongly associated variant for cycle length is rs564036233. LD (1000 Genomes Nov 2014 EUR) shown is with rs10835638, the *FSHB* promoter polymorphism. Other SNPs indicated were the variants most significantly associated with FSH (rs11031005) and LH (rs11031002) in a GWAS of hormone levels (Ruth *et al.*, 2015), and with age at natural menopause (rs12294104) in a meta-analysis (Stolk *et al.*, 2012). *KCNA4*: potassium channel, voltage-gated shaker-related subfamily A, member 4. *ARL14EP*: ADP-ribosylation factor-like GTPase 14 effector protein. *MPPED2*: metallophosphoesterase domain containing 2. Note: LD values are not available for all SNPs since they are not included in 1000 Genomes Nov 2014 EUR. Position is in build hg19/GRCh37.

Variants in or near the *FSHB* gene were the only ones that reached a conservative level of genome-wide significance in the GWAS for menstrual cycle length (Fig. 2). The strongest association was for rs564036233G>GA, a 1 bp insertion which was associated with longer cycles by 1 day (0.16 SD) per minor allele (95% CI 0.12–0.20; *P* = 1.30 × 10^−16^). The rs564036233 variant is in strong linkage disequilibrium (LD) with the promoter polymorphism rs10835638 (*r*^2^ = 0.82) and conditional analysis indicated that rs564036233 and rs10835638 represent the same signal. The next strongest signal in the GWAS was on Chromosome 9 in the *NOTCH1* gene, but did not meet our genome-wide significance threshold and would require further replication (rs3124592A>G; MAF 0.45; 0.08 SD per minor allele; 95% CI 0.05–0.11; *P* = 1.9 × 10^−8^).

### The FSHB allele associated with longer cycle length is associated with later menopause

The FSH-lowering T allele of rs10835638 was associated with later age at menopause for those in the UK Biobank [0.13 years per minor allele (*ReproGen* definition); 95% CI 0.04–0.22; *P* = 5.7 × 10^−3^]. There was no association between rs10835638 and menopause age when we dichotomized the phenotype into early menopause compared with later menopause (Table III). The *FSHB* locus is known to be associated with timing of menopause: in a GWAS conducted by the *ReproGen* consortium, the signal at this locus (rs12294104) increases age at menopause by 0.23 years (95% CI 0.16–0.29; *P* = 1.5 × 10^−11^) (Stolk *et al.*, 2012). Later menopause has been shown to be associated with later age at last birth (Ayatollahi *et al.*, 2003; Dorjgochoo *et al.*, 2008) and rs10835638 was also associated with later age at last birth [0.02 SD (∼0.1 years) per T allele; 95% CI 0.00–0.04; *P* = 4.2 × 10^−2^].

### Longer cycle length is not a general feature of alleles associated with later age at menopause

**Figure 3:**
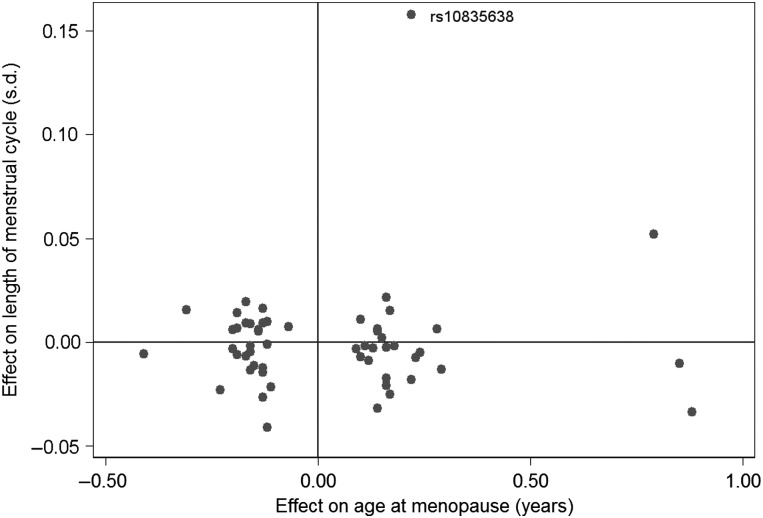
Comparison of the published effect size of the 56 known age at menopause variants (Stolk *et al.*, 2012; Perry *et al.*, 2014a,b) and their effect size in the GWAS for menstrual cycle length. There was no significant correlation between the effects on age at menopause and cycle length (*R* = 0.064, *P* = 0.63). The *FSHB* promoter polymorphism (rs10835638) is indicated.

We next tested the role of all 56 genetic variants associated with age at menopause. In addition to the age at menopause signal at the *FSHB* locus (rs12294104), only one of the other 55 published age at menopause signals was nominally associated with cycle length (*P* > 0.05): rs10734411 was associated at *P* = 0.005 (Stolk *et al.*, 2012; Perry *et al.*, 2014a,b; Day *et al.*, 2015). For the 56 published menopause SNPs, there was no correlation between the published effect estimates for age at menopause and the effect estimates from our GWAS for menstrual cycle length (*R* = 0.064, *P* = 0.63) (Fig. 3). The *FSHB* SNP was an outlier in this plot, but removing it did not substantially affect the correlation (*R* = −0.027; *P* = 0.84).

### The FSHB allele associated with lower FSH is also associated with an indicator of female infertility

The FSH-lowering T allele of the *FSHB* promoter polymorphism (rs10835638) was associated with female nulliparity, i.e. greater odds of never being pregnant (OR = 1.06; CI 1.02–1.11*; P* = 4.8 × 10^−3^) (Fig. 1). The FSH-lowering allele was not associated with other possible indicators of female infertility (later age at first birth and fewer live births) or male infertility (number of children fathered) (*P* > 0.05) (Table III).

### The FSHB allele associated with higher FSH is also associated with higher odds of endometriosis and surgical intervention

The more common G allele was associated with increased odds of endometriosis (OR = 1.27; CI 1.11–1.45; *P* = 4.1 × 10^−4^) (Fig. 1). Of the seven published GWAS variants associated with endometriosis risk (Nyholt *et al.*, 2012), the variant on chromosome 12 was nominally associated with cycle length, with the allele associated with an increased risk of endometriosis also associated with shorter cycles (*P* = 0.02).

The G allele of rs10835638 was also associated with increased odds of having the medical interventions bilateral oophorectomy (OR = 1.12; 95% CI 1.06–1.19; *P* = 1.4 × 10^−4^) and hysterectomy (OR = 1.13; 95% CI 1.06–1.20; *P* = 1.0 × 10^−4^), which are used as treatments for a range of gynaecological conditions including endometriosis.

### The common FSHB variant, associated with FSH levels, is not associated with reproductive traits more generally

There was no consistent evidence that the *FSHB* variant (rs10835638) was associated with age at menarche. There was a 0.03-year increase in age at menarche (*ReproGen* definition) per T allele of rs10835638 (95% CI 0.01–0.05; *P* = 1.4 × 10^−2^) and the binary phenotype of early menarche was associated at *P* > 0.05 (Table III). None of 122 published GWAS signals for menarche (Perry *et al.*, 2014a,b) were associated with length of menstrual cycle at *P* < 0.008.

The *FSHB* promoter polymorphism (rs10835638) was not associated with other reproductive illnesses or conditions at *P* < 0.05 (Table III), except for menopausal symptoms (OR = 0.62; 95% CI 0.41–0.93; *P* = 0.02) (Fig. 1). No associations were found with dysmenorrhoea, fibroids, irregular menstrual cycles, menorrhagia, multiple pregnancy loss, ovarian cysts, PCOS, uterine polyps or vaginal/uterine prolapse, or with female breast, ovarian or endometrial cancer.

## Discussion

In the first GWAS of menstrual cycle length, we found a strong association between an FSH lowering, likely functional, variant in the *FSHB* promoter and longer cycles (Hoogendoorn *et al.*, 2003; Grigorova *et al.*, 2008, 2010; Tuttelmann *et al.*, 2012; Benson *et al.*, 2013; La Marca *et al.*, 2013; Simoni and Casarini, 2014; Ruth *et al.*, 2015). This locus has been previously robustly associated with age at menopause in the *ReproGen* consortium GWAS of menopause timing (Stolk *et al.*, 2012; Day *et al.*, 2015) and the allele associated with longer cycle length is also associated with later age at menopause. We did not observe associations for the majority of age at menopause GWAS signals with length of menstrual cycle, including the four signals with effects of over one-third of a year per allele on menopause timing, implying that the association is specific to *FSHB:* either FSH-β has independent effects on both cycle length and menopause or changes in cycle length are causally influencing menopause timing.

Our results are consistent with the observed epidemiological relationship between longer menstrual cycles and later age at menopause (Whelan *et al.*, 1990; Kaczmarek, 2007). It is possible that there is a biological limit on the lifetime number of menstrual cycles; hence, women with longer cycles would have later menopause. Alternatively, they may have reduced follicle recruitment per cycle, depleting their ovarian reserve more slowly. Women with longer cycles have more waves of folliculogenesis during each cycle (Baerwald *et al.*, 2003, 2012) but may recruit fewer antral follicles per wave. Oocyte loss due to ovulation is unlikely to be driving the relationship, since this contributes much less to overall oocyte depletion than atresia, and there is no robust evidence that preventing ovulation by the use of the combined oral contraceptive pill influences menopause timing (van Noord *et al.*, 1997; de Vries *et al.*, 2001; Gold *et al.*, 2001, 2013; Ayatollahi *et al.*, 2003; Palmer *et al.*, 2003; Kaczmarek, 2007; Dorjgochoo *et al.*, 2008; OlaOlorun and Lawoyin, 2009; Pokoradi *et al.*, 2011; Stepaniak *et al.*, 2013) and both longer and shorter cycles are more likely to be anovulatory (Mihm *et al.*, 2011). More work is needed to understand the molecular mechanism that explains the association between cycle length and menopause timing.

The FSH-reducing allele was associated with nulliparity, perhaps indicating increased female infertility. Although we were unable to distinguish those unable to have children from those not wishing to, the sample of nulliparous women will be enriched for both female and male factor infertility. The FSH-lowering allele has previously been found to be associated with male infertility (Grigorova *et al.*, 2008, 2010; Tuttelmann *et al.*, 2012; Simoni and Casarini, 2014), but we found no association with males who had never fathered a child suggesting a female-specific effect, although this may because the phenotype includes males who chose not to have children in addition to infertile males. Using nulliparity as a proxy for infertility is unlikely to generate a false-positive association, but may have reduced our power to detect a true association. The relationship between FSH and fertility over a woman's lifetime may differ from the age-related changes in FSH around menopause. In contrast to our genetic association between lower FSH and infertility, women nearing menopause have higher FSH concentrations, poorer ovarian reserve and decreased fertility (Waller *et al.*, 1998; Mihm *et al.*, 2011). FSH is required for follicle development and it is proposed that an FSH threshold is required to achieve ovulation (Kumar *et al.*, 1997, 1999). Ovulation increases with increasing FSH in transgenic mice with FSH levels that increase with age independently of follicle depletion (McTavish *et al.*, 2007). A high baseline level of FSH, determined by genetic variation, may promote ovulation and explain our association with parity.

The FSH-increasing allele increased the risk of endometriosis in our study. Several GWAS of endometriosis have been performed; however, none have reported a signal at the 11p14.1 locus and there was no evidence that the genome-wide significant endometriosis variants were associated with cycle length in our study (Adachi *et al.*, 2010; Uno *et al.*, 2010; Painter *et al.*, 2011; Nyholt *et al.*, 2012; Albertsen *et al.*, 2013). Drug treatments for endometriosis aim to prevent ovulation and menstruation, and to stabilize hormone levels, since estrogens fuel ectopic endometrial growth (Vercellini *et al.*, 2014). The FSH-increasing allele may similarly stimulate abnormal growth of endometrium. Endometriosis is associated with earlier menopause (Pokoradi *et al.*, 2011; Yasui *et al.*, 2011) and shorter menstrual cycles (Vercellini *et al.*, 2014), consistent with our findings. The FSH-increasing variant associated with increased risk of endometriosis was also associated with parity; however, endometriosis can cause infertility as a result of endometriotic lesions and chronic pelvic inflammation. Therefore, the association of the *FSHB* polymorphism with infertility appears to be independent of the association with endometriosis.

We found a modest association of the FSH-lowering allele with increased age at menarche, but the published age at menarche GWAS signals were not associated with length of menstrual cycle. The closest GWAS menarche signal to *FSHB* (rs16918636) is 1.13 Mb away and is not in LD (*r*^2^ = 0.001) with the *FSHB* promoter polymorphism SNP (Perry *et al.*, 2014a,b). Although FSH is important for normal puberty, the role of variation in baseline FSH levels on puberty timing is uncertain.

The UK Biobank recruited individuals over 40 years old, and many of the women still cycling will be approaching menopause; however, if the association with cycle length was being driven by peri-menopausal changes, we would expect all menopause-associated variants to be associated with cycle length. In addition, our sensitivity analysis suggested a stronger effect of the *FSHB* promoter polymorphism in younger women. We were unable to replicate an association between the FSH-lowering allele and increased odds of PCOS (Hayes *et al.*, 2015). However, we had only a small number of cases (*n* = 153) limiting our power to detect this association. Other illnesses had relatively small sample sizes and may have been similarly under-powered. We might have also under-ascertained cases, as most illnesses will be subject to recall bias as they are self-reported and collected retrospectively, while controls might include people not reporting an illness.

Our study provides evidence that a likely functional variant in the *FSHB* promoter is strongly associated with longer menstrual cycles, and to a lesser extent with female infertility and lower risk of endometriosis. There is considerable evidence that the T allele of the *FSHB* promoter polymorphism decreases FSH levels (Hoogendoorn *et al.*, 2003; Grigorova *et al.*, 2008, 2010; Tuttelmann *et al.*, 2012; Benson *et al.*, 2013; La Marca *et al.*, 2013; Simoni and Casarini, 2014; Ruth *et al.*, 2015), but it has also been associated with increased LH levels (Hayes *et al.*, 2015; Ruth *et al.*, 2015). While we cannot rule out that the variant may be having direct or indirect effects on other hormone levels, a change in FSH is the most likely primary mechanism. In conclusion, we suggest that lower FSH levels result in longer menstrual cycles and as a consequence later menopause and, while having detrimental effects on female fertility, are protective against endometriosis.

## Supplementary data

Supplementary data are available at http://humrep.oxfordjournals.org/.

## Authors' roles

A.M. and K.S.R. designed the study, carried out analysis and drafted the article. All authors were involved in designing and performing analysis of the UK Biobank data, revising and approving the manuscript.

## Funding

A.R.W., H.Y. and T.M.F. are supported by the European Research Council grant: 323195:GLUCOSEGENES-FP7-IDEAS-ERC. R.M.F. is a Sir Henry Dale Fellow (Wellcome Trust and Royal Society grant: 104150/Z/14/Z). R.N.B. is funded by the Wellcome Trust and Royal Society grant: 104150/Z/14/Z. J.T. is funded by the ERDF and a Diabetes Research and Wellness Foundation Fellowship. S.E.J. is funded by the Medical Research Council (grant: MR/M005070/1). M.A.T., M.N.W. and A.M. are supported by the Wellcome Trust Institutional Strategic Support Award (WT097835MF) (323195). The funders had no influence on study design, data collection and analysis, decision to publish or preparation of the manuscript. Funding to pay the Open Access publication charges for this article was provided by The Wellcome Trust.

## Conflict of interest

None declared.

## Supplementary Material

Supplementary Data
