# Supplementary material for: Genetic evidence that lower circulating FSH levels lengthen menstrual cycle, increase age at menopause and impact female reproductive health
Source: Hum Reprod. 2016 Jan 4;31(2):473–81. doi: 10.1093/humrep/dev318 (PMC4716809; doi:10.1093/humrep/dev318)
Supplement: Supplementary Data [file supp_31_2_473__index.html]

Genetic evidence that lower circulating FSH levels lengthen menstrual cycle, increase age at menopause and impact female reproductive health — Genetic evidence that lower circulating FSH levels lengthen menstrual cycle, increase age at menopause and impact female reproductive health — Supplementary Data 

# Genetic evidence that lower circulating FSH levels lengthen menstrual cycle, increase age at menopause and impact female reproductive health

## Supplementary Data

Supplementary Data

- Supplementary Data - pdf file
- Supplementary Figure 1 - pdf file
- Supplementary Figure 2 - pdf file
- Supplementary Figure 3 - pdf file
- Supplementary Table 1 - pdf file
