# Supplementary material for: Genetic evidence that lower circulating FSH levels lengthen menstrual cycle, increase age at menopause and impact female reproductive health
Source: Hum Reprod. 2016 Jan 4;31(2):473–81. doi: 10.1093/humrep/dev318 (PMC4716809; doi:10.1093/humrep/dev318)
Supplement: Supplementary Data [file supp_dev318_dev318supp_data.pdf]

Human Reproduction
doi:10.1093/humrep/dev318

Definitions of the reproductive traits used in the analysis

Reproductive trait	Definition
Age at first birth (years)	Age at first live birth (females only)
Age at last birth (years)	Age at last live birth (females only)
Age at menarche (years)	Age at menarche between 9 and 17 years
Age at natural menopause (years)	Age at last menstrual period excluding those with surgical menopause or taking hormone replacement therapy
Bilateral oophorectomy	Ever had bilateral oophorectomy (case) versus never (control)
Breast cancer	Breast cancer on registry (ICD10 C50, ICD9 174 and 175), versus no cancer reported (control)
Dysmenorrhoea	Dysmenorrhoea listed as an illness (reported at interview)
Early menarche	Youngest 5% of BMI adjusted age at menarche (case) versus oldest 5% (control). Age at menarche defined as above
Early menopause	Age at natural menopause (as defined above) at 20–45 years (case) versus 50–60 years (control)
Endometrial cancer	Endometrial cancer on registry (ICD10 C54, ICD9 182) versus no cancer reported
Endometriosis	Endometriosis listed as an illness (reported at interview)
Fibroids	Fibroids listed as an illness (reported at interview)
Hysterectomy	Ever had hysterectomy (case) versus never (control)
Irregular menstrual cycles	Women still menstruating reporting irregular cycles (case) versus regular cycles (control)
Length of menstrual cycle (days)	Women still menstruating reporting regular cycles. Excludes women taking oral contraceptives, HRT or hormone medications (see list below) and pregnant women
Long menstrual cycle (versus average)	Length of menstrual cycle >31 days (case) versus 28 days (control). As defined above
Menopausal symptoms	Menopausal symptoms listed as an illness (reported at interview)
Menorrhagia	Menorrhagia listed as an illness (reported at interview)
Multiple pregnancy loss	Cases are women with two or more pregnancies lost due to still births and miscarriages. Controls are women who have had a live birth and have never had a stillbirth or miscarriage
Never fathered child	Never fathered a child (case) versus one or more children fathered
Never pregnant	Never pregnant (case) versus one or more pregnancies (control). Number of pregnancies calculated from total number live births, still births, miscarriages, terminations
Number of children fathered	Number of children fathered. Males only
Number of live births	Number of live births. Females only
Ovarian cancer	Ovary cancer on registry (ICD10 C56, ICD9 183) (case) versus no reported cancer (control)
Ovarian cysts	Ovarian cysts listed as an illness (reported at interview)
Polycystic ovary syndrome (PCOS)	Polycystic ovary syndrome listed as an illness (reported at interview)
Short menstrual cycle (versus average)	Length of menstrual cycle ≤ 20 days (case) versus 28 days (control). As defined above
Uterine polyps	Uterine polyps listed as an illness (reported at interview)
Vaginal/uterine prolapse	Vaginal/uterine prolapse listed as an illness (reported at interview)

Medications that resulted in exclusion from the length of menstrual cycle analyses

UK Biobank medication code	UK Biobank description
1141182800	Cerazette 75 µg tablet
1140869346	Cilest tablet
1140864196	Climagest 1 mg tablet
1141172714	Climanor 5 mg tablet
1140868372	Climaval 1 mg tablet
1140926430	Climesse tablet
1141180944	Clomifene
1140884638	Clomifene
1140868406	Conjugated estrogens
1141190580	Conjugated estrogens 0.3 mg/medroxyprogesterone 1.5 mg tab
1141152356	Cyclogest 200 mg pessary
1140868590	Cyclogest 200 mg suppository
1140868508	Cyclo-progynova 1 mg tablet
1140884634	Cyproterone
1141192344	Cyproterone acetate + ethinylestradiol
1140876638	Cyproterone acetate + ethinyloestradiol
1140868968	Danazol
1141157298	Danazol product
1140857620	Depo-provera 50 mg/1 ml injection
1140857912	Desogestrel
1141182794	Desogestrel product
1140880234	Dianette tablet
1141156644	Elleste duet conti tablet
1140923852	Elleste-solo 1 mg tablet
1141152228	Elleste-solo mx 40 patch
1140926592	Estraderm mx 25 patch
1141181700	Estradiol product
1141181818	Estradiol + norethisterone acetate 1 mg/0.5 mg tablet
1141202030	Estradot 25 µg patch
1140868470	Estrapak 50 µg/1 mg patch + tablet
1140911708	Estring 2 mg(7.5 µg/24 h) vaginal ring
1141181594	Estriol product
1141181220	Ethinylestradiol
1141181218	Ethinylestradiol product
1141181286	Ethinylestradiol + desogestrel 20 µg/150 µg tablet
1141179822	Ethinylestradiol + drospirenone 30 µg/3 mg tablet
1141181306	Ethinylestradiol + gestodene 20 µg/75 µg tablet
1141181240	Ethinylestradiol + levonorgestrel 30 µg/150 µg tablet
1141192874	Ethinylestradiol + norelgestromin 600 µg/6 mg transdermal patch
1141181298	Ethinylestradiol + norethisterone acetate 20 µg/1 mg tablet
1141181204	Ethinylestradiol + norgestimate 35 µg/250 µg tablet
1140910674	Ethinylnortestosterone
1140868446	Ethinyloestradiol
1141157404	Ethinyloestradiol product

Continued

Continued

UK Biobank medication code	UK Biobank description
1140869166	Ethinyloestradiol + desogestrel 20 µg/150 µg tablet
1140869172	Ethinyloestradiol + ethynodiol diacetate 30 µg/2 mg tablet
1141166366	Ethinyloestradiol + gestodene 20 µg/75 µg tablet
1140869248	Ethinyloestradiol + levonorgestrel 30 µg/150 µg tablet
1140869328	Ethinyloestradiol + norethisterone acetate 20 µg/1 mg tablet
1140869348	Ethinyloestradiol + norgestimate 35 µg/250 µg tablet
1141166196	Etonogestrel
1140916790	Evorel 25 patch
1141151718	Evorel conti patch
1141192876	Evra transdermal patch
1141145900	Femara 2.5 mg tablet
1140923598	Fematrix 40 patch
1140869334	Femodene tablet
1141166368	Femodette tablet
1140922562	Femoston 1/10 tablet
1140923738	Femseven 50 patch
1141193320	Femtab 1 mg tablet
1141193318	Femtab continuous tablet
1141193316	Femtab sequi tablet
1140869362	Femulen tablet
1140868882	Gonadorelin
1140909920	Gonadotrophin-releasing hormone product
1140870194	Goserelin
1141157394	Goserelin product
1141184648	Human luteinizing hormone product
1140882962	Human menopausal gonadotrophins
1141166200	Implanon 68 mg subdermal implant
1141172722	Levonelle 750 µg tablet
1140869366	Levonorgestrel
1141157410	Levonorgestrel product
1140869162	Marvelon tablet
1140858324	Medroxyprogest 80 mg/ml suspension 100 ml
1140869270	Medroxyprogesterone
1141177658	Menopur 75 iu injection (pdr for recon) + solvent
1140884626	Mestranol
1141157492	Mestranol product
1140869356	Mestranol + norethisterone 50 µg/1 mg tablet
1140869180	Microgynon 30 tablet
1140869276	Micronor tablet
1140869112	Mifepristone
1141157302	Mifepristone product
1140921822	Mirena 20 µg/24 h intrauterine system
1140921814	Mirena 52 mg intrauterine system
1140868580	Norethisterone
1141157406	Norethisterone product
1140869370	Norgeston tablet
1140869278	Noriday tablet

Continued

Continued

UK Biobank medication code	UK Biobank description
1140869260	Norimin tablet
1140917448	Estradiol 1.25 g/dose gel
1140857700	Estradiol 1 mg/1 ml injection
1140857690	Estradiol 25 mg implant 36 week
1140868456	Estradiol product
1141168324	Estradiol + norethisterone acetate 1 mg/0.5 mg tablet
1140870186	Oestrifen 10 mg tablet
1140857706	Oestriol 250 μg tablet
1140868400	Oestriol product
1141167206	Oestrogel 0.06% gel
1140917450	Oestrogel 1.25 g gel
1140884622	Estrogen product
1140869186	Ovranette tablet
1140869262	Ovysmen tablet
1140868408	Premarin 625 μg tablet
1140922804	Premique 0.625 mg/5 mg tablet
1140922806	Premique cycle 10 mg tablet
1140857636	Prempak 0.625 tablet
1140868588	Progesterone product
1140868460	Progynova 1 mg tablet
1141180580	Progynova ts 50 50 μg patch
1140923914	Progynova ts 50 μg patch
1140870284	Prostap sr 3.75 mg injection (pdr for recon) + diluent + kit
1140869190	Trinordiol tablet
1140869266	Trinovum tablet
1140868514	Trisequens tablet
1141179824	Yasmin tablet
