# Supplementary figures and images for: Genetic evidence that lower circulating FSH levels lengthen menstrual cycle, increase age at menopause and impact female reproductive health
Source: Hum Reprod. 2016 Jan 4;31(2):473–81. doi: 10.1093/humrep/dev318 (PMC4716809; doi:10.1093/humrep/dev318)

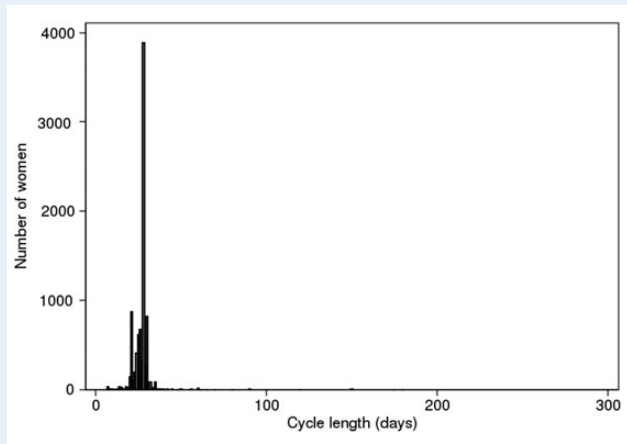

**Supplementary Figure S1** Length of menstrual cycle (all).

Supplement: Supplementary Data [file supp_dev318_dev318supp_fig1.pdf]

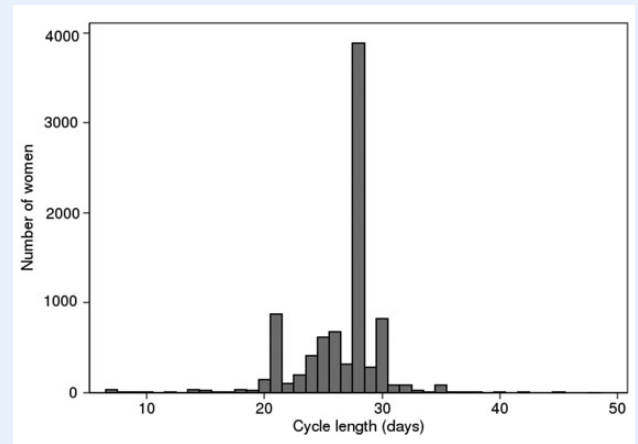

**Supplementary Figure S2** Length of menstrual cycle (cycle length under 50 days).

Supplement: Supplementary Data [file supp_dev318_dev318supp_fig2.pdf]
