# Supplementary material for: Genetic evidence that lower circulating FSH levels lengthen menstrual cycle, increase age at menopause and impact female reproductive health
Source: Hum Reprod. 2016 Jan 4;31(2):473–81. doi: 10.1093/humrep/dev318 (PMC4716809; doi:10.1093/humrep/dev318)
Supplement: Supplementary Data [file supp_dev318_dev318supp_fig3.pdf]

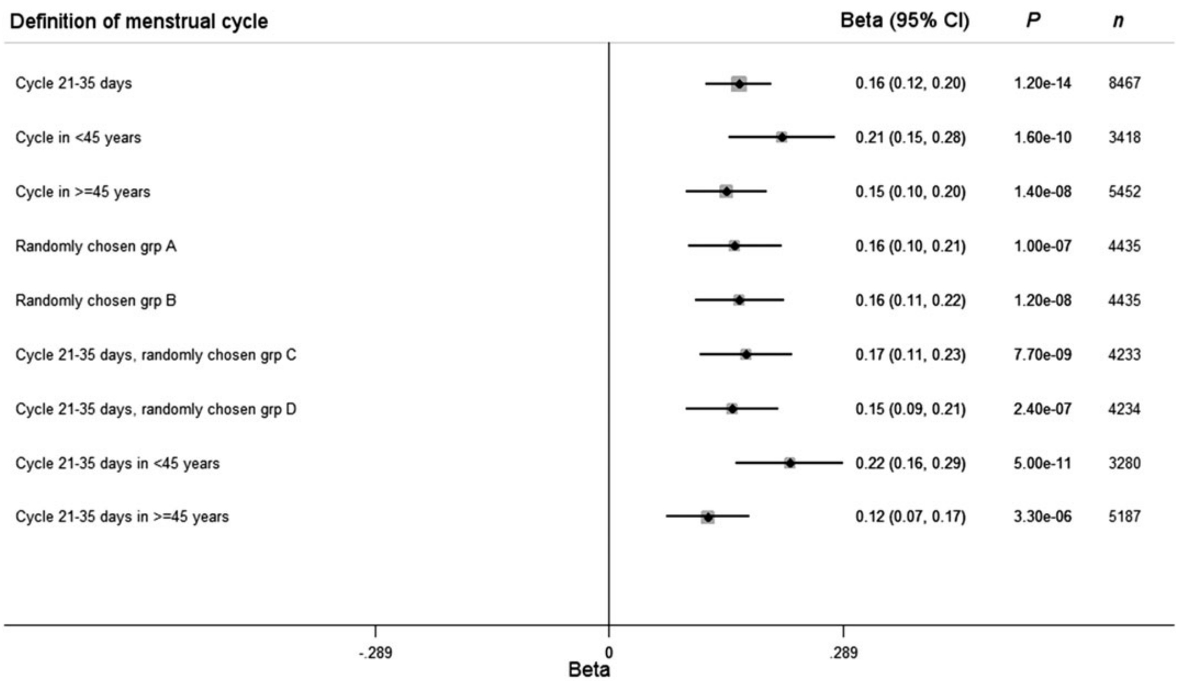

Length of menstrual cycle (days)

	N	Min	Max	Mean	S.D.	Lower quartile	Median	Upper quartile
Cycle 21-35 days	8,467	21	35	26.9	2.8	25	28	28
Cycle in <45 years	3,418	7	175	26.7	4.7	25	28	28
Cycle >=45 years	5,452	7	300	26.9	6.9	25	28	28
Randomly chosen grp A	4,435	7	175	26.8	5.4	25	28	28
Randomly chosen grp B	4,435	7	300	26.9	6.9	25	28	28
Cycle 21-35 days, randomly chosen grp C	4,233	21	35	26.8	2.9	25	28	28
Cycle 21-35 days, randomly chosen grp D	4,234	21	35	26.9	2.7	25	28	28
Cycle 21-35 days in <45 years	3,280	21	35	26.9	2.8	25	28	28
Cycle 21-35 days in >=45 years	5,187	21	35	26.9	2.8	25	28	28

Supplementary Figure S3 Results of sensitivity analyses for length of menstrual cycle. *Notes*: Sensitivity analyses were carried out by restricting the analysis to women with menstrual cycles from 21 to 35 days; conducting the analysis in women aged under 45 or 45 years and older; and carrying out the analysis in a split sample of two groups of equal size randomly selected from the full cohort. Effects (β) are in standard deviations of the inverse-normally transformed variable. grp, group.
