# Supplementary material for: Genetic evidence that lower circulating FSH levels lengthen menstrual cycle, increase age at menopause and impact female reproductive health
Source: Hum Reprod. 2016 Jan 4;31(2):473–81. doi: 10.1093/humrep/dev318 (PMC4716809; doi:10.1093/humrep/dev318)
Supplement: Supplementary Data [file supp_dev318_dev318supp_table1.pdf]

Supplementary Table SI **Age at recruitment and cycle length for women included in analysis of length of menstrual cycle.**

Phenotype	Analysis	*n*	Min	Max	Mean	SD	Lower quartile	Median	Upper quartile
Length of menstrual cycle (days)	FSHB promoter polymorphism analysis	8870	7	300	26.8	6.2	25	28	28
Length of menstrual cycle (days)	GWAS	9534	7	300	26.8	6.1	25	28	28
Age at recruitment (years)	FSHB promoter polymorphism analysis	8870	40	68	45.9	3.7	43	46	48
Age at recruitment (years)	GWAS	9534	40	68	45.9	3.7	43	46	48

The GWAS analysis included genetically related individuals excluded from the analysis of the association of the *FSHB* promoter polymorphism resulting in an increased total *n*.
